# Atmosphere Effect in Post-Annealing Treatments for Amorphous InGaZnO Thin-Film Transistors with SiO_x_ Passivation Layers

**DOI:** 10.3390/mi12121551

**Published:** 2021-12-12

**Authors:** Wen Zhang, Zenghui Fan, Ao Shen, Chengyuan Dong

**Affiliations:** Department of Electronic Engineering, Shanghai Jiao Tong University, Shanghai 200240, China; wen_zhang0032@sjtu.edu.cn (W.Z.); zenghui_f@sjtu.edu.cn (Z.F.); shenao1997@sjtu.edu.cn (A.S.)

**Keywords:** amorphous InGaZnO (a-IGZO), thin-film transistor (TFT), positive bias stress (PBS), annealing atmosphere, oxygen vacancy

## Abstract

We investigated the electrical performance and positive bias stress (PBS) stability of the amorphous InGaZnO thin-film transistors (a-IGZO TFTs) with SiO_x_ passivation layers after the post-annealing treatments in different atmospheres (air, N_2_, O_2_ and vacuum). Both the chamber atmospheres and the device passivation layers proved important for the post-annealing effects on a-IGZO TFTs. For the heat treatments in O_2_ or air, the larger threshold voltage (V_TH_) and off current (I_OFF_), smaller field-effect mobility (μ_FE_), and slightly better PBS stability of a-IGZO TFTs were obtained. The X-ray photoemission spectroscopy (XPS) and secondary ion mass spectroscopy (SIMS) measurement results indicated that the oxygen atoms from the ambience led to less oxygen vacancies (V_O_) and more oxygen-related defects in a-IGZO after the heat treatments in O_2_ or air. For the annealing processes in vacuum or N_2_, the electrical performance of the a-IGZO TFTs showed nearly no change, but their PBS stability evidently improved. After 4500 seconds’ stressing at 40 V, the V_TH_ shift decreased to nearly 1 V. In this situation, the SiO_x_ passivation layers were assumed to effectively prevent the oxygen diffusion, keep the V_O_ concentration unchanged and refuse the oxygen-related defects into the a-IGZO films.

## 1. Introduction

Amorphous InGaZnO thin-film transistors (a-IGZO TFTs) have been regarded as among the most promising active-matrix devices for next-generation flat panel displays (FPDs) due to their high mobility, good uniformity and low fabrication temperature [1,2,3,4,5]. For mass productions of a-IGZO TFTs, either etching-stopper (ES) or back-channel-etching (BCE) structures are used. Although the better electrical performance and stability are obtained for ES-structured devices, BCE is still preferred for its simpler processing and compatibility with amorphous silicon (a-Si) TFT productions [6,7,8,9]. Hence, researchers have been taking many measures to improve the electrical performance and stability of BCE-structured a-IGZO TFTs, among which post-annealing treatments seem extremely useful [10,11]. Some studies pointed out that the annealing effect was closely associated with the oxygen vacancy (V_O_), one of the most important defects in a-IGZO films [12,13]. Assuming V_O_ was sensitive to annealing atmospheres, as some researchers obtained higher performances and stabler properties of a-IGZO TFTs by choosing appropriate annealing atmospheres (e.g., oxygen, nitrogen, vacuum, and so on) [14,15,16,17]. However, all of these studies were limited to the unpassivated devices whereas the TFT devices are always passivated in mass production. Since passivation layers might exhibit different effects under different annealing atmospheres, it might be meaningful to investigate the atmosphere effect in post-annealing treatments for the a-IGZO TFTs with passivation layers.

In this study, the electrical performance and positive bias stress (PBS) stability of the a-IGZO TFTs with SiO_x_ passivation layers under various post-annealing atmospheres (air, O_2_, N_2_ and vacuum) were comparatively investigated, whose physical essence was deeply analyzed with the help of X-ray photoemission spectroscopy (XPS) and secondary ion mass spectroscopy (SIMS). We found that both the chamber atmospheres and the device passivation layers played important roles in the post-annealing treatments for a-IGZO TFTs. A qualitative model was also built to explain the experimental results.

## 2. Materials and Methods

**Figure 1:**
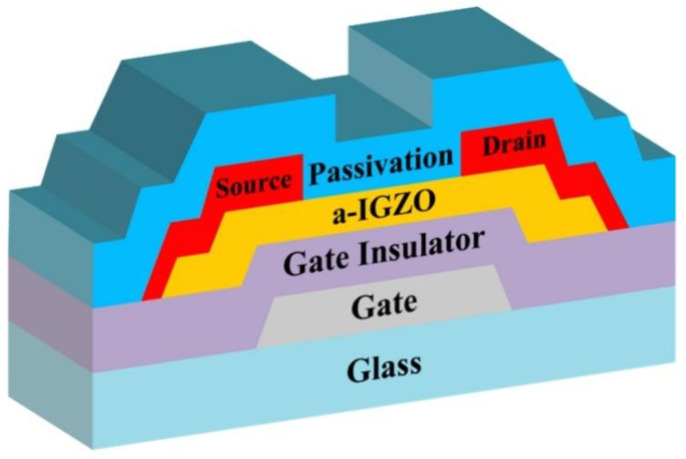
Schematic cross-section of the back-channel-etching (BCE) -structured amorphous InGaZnO thin-film transistors (a-IGZO TFTs) in this study.

Figure 1 shows the schematic cross-section of the BCE-structured a-IGZO TFTs used in this study. Firstly, the gate electrodes (150 nm-thick aluminum and 23 nm-thick molybdenum films) were deposited on the glass substrates by sputtering. Then, 350 nm-thick SiN_x_ and 50 nm-thick SiO_x_ films were prepared as gate insulators (GIs) by plasma-enhanced chemical vapor deposition (PECVD). The 60 nm-thick channel layers were deposited by RF sputtering at room temperature (RT) using an IGZO target, where the atoms ratio of In:Ga:Zn was 1:1:1. After patterning the channel layers, a pre-annealing treatment (300 °C/1 h/air) was employed in a furnace, which could increase the film density/uniformity and reduce the defects in the a-IGZO films. The 30 nm-thick molybdenum and 150 nm-thick aluminum films were then prepared and patterned as source/drain (S/D) electrodes before the 200 nm-thick SiO_x_ films were deposited as passivation layers by PECVD. Finally, the samples were annealed at 300 °C for 1 h in different atmospheres (air, O_2_, N_2_ and vacuum) using a rapid thermal processing (RTP) oven, where the gas flows of O_2_ (or N_2_) were set to 1 L/min and the vacuum pressure was kept at 6.5 × 10^−3^ Pa. It is worth noting that all the films were patterned by standard lithography and etching processes. The typical channel width/length of the a-IGZO TFTs was 12/6 μm.

The electrical performance and PBS stability of the a-IGZO TFTs were measured by a Keithley 2636 analyzer. The transfer curves were obtained when V_GS_ was scanned from −20 V to 40 V at a step of 0.5 V and V_DS_ was set to 10 V. During the PBS tests, the transfer curves were instantly measured following each 1500 s’ stressing (V_GS_ = +40 V). All the above tests were performed in a dark chamber at RT. An XPS analyzer (AXIS Ultra DLD) was used to characterize the chemical bonding states of the a-IGZO films (without or with passivation layers) annealed under different atmospheres. Here, an X-ray source with the aluminum anode was used to bombard the film surface and obtain the energy spectrum of different elements. The binding energy of all elements were calibrated using the C1s peak at 284.8 eV. A SIMS measurement system (TOF SIMS 5 produced by ION-TOF GmbH) with a Cs^+^ primary ion source was applied to measure the depth profiles of different elements.

## 3. Results and Discussion

**Figure 2:**
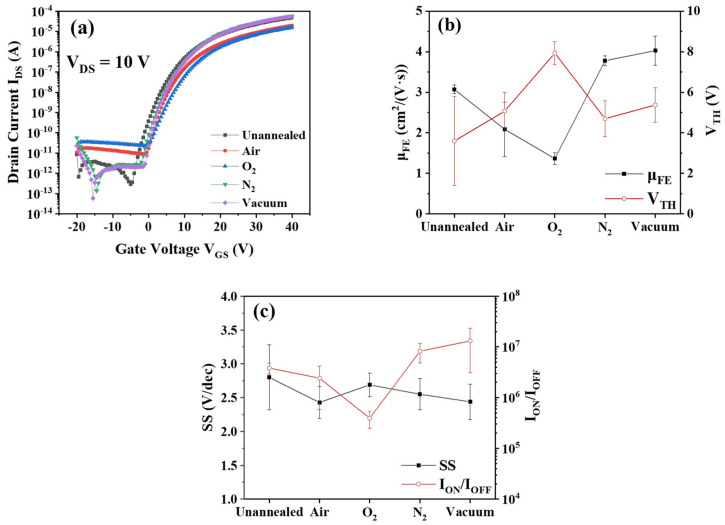
(**a**) Transfer curves; (**b**) mobility/threshold voltage; and (**c**) sub-threshold slope/on–off current ratio of the a-IGZO TFTs with SiO_x_ passivation layers annealed in different atmospheres.

Figure 2a shows the transfer curves of the a-IGZO TFTs annealed under different atmospheres, where the unannealed device was considered the reference sample. The corresponding performance parameters including threshold voltage (V_TH_), field-effect mobility (μ_FE_), sub-threshold swing (SS) and on–off current ratio (I_ON_/I_OFF_) were extracted from the transfer curves according to Ref. [18]. Here, 6–12 devices were measured and statistically analyzed. As shown in Figure 2b, the average mobilities of the devices annealed under different atmospheres (unannealed, air, O_2_, N_2_ and vacuum) were 3.07, 2.08, 1.37, 3.78 and 4.03 cm^2^/(V·s), respectively. One may notice that the annealing in O_2_ or air obviously degraded the device mobility, whereas the annealing in N_2_ or vacuum slightly improved μ_FE_. In addition, the corresponding V_TH_ values were 3.6, 5.1, 8.0, 4.7 and 5.4 V, the SS values were 2.8, 2.4, 2.7, 2.6 and 2.4 V/dec, and the on–off current ratios were 3.8 × 10^6^, 2.4 × 10^6^, 3.9 × 10^5^, 8.2 × 10^6^ and 1.3 × 10^7^, respectively (see Figure 2b,c). Compared with the unannealed sample, one may observe from Figure 2b that the post-annealing in O_2_ evidently increased the threshold voltages of a-IGZO TFTs, while the annealing treatments in N_2_ or vacuum showed nearly no influence on the V_TH_ values. As shown in Figure 2c, the SS values of a-IGZO TFTs remained almost the same under various post-annealing atmospheres. The post-annealing treatments resulted in sufficiently large on/off current ratios (>10^6^) except that in O_2_, as shown in Figure 2c.

**Figure 3:**
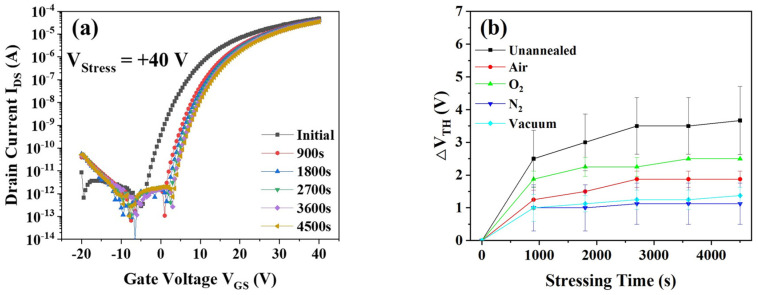
(**a**) Stressing time dependence of the transfer curves of the unannealed a-IGZO TFTs with SiO_x_ passivation layers; (**b**) threshold voltage shifts of the a-IGZO TFTs with SiO_x_ passivation layers annealed under different atmospheres during PBS tests.

The bias stress effect plays an important role in the actual applications of TFT devices [2,19]. Accordingly, we measured the PBS stability of the a-IGZO TFTs with SiO_x_ passivation under various annealing atmospheres (unannealed, air, O_2_, N_2_ and vacuum). Figure 3a shows the transfer curve evolution of the unannealed a-IGZO TFTs during PBS tests. With the stressing time elapsed, the transfer curves of the devices shifted positively. In order to quantitatively describe this unstable property, we defined a useful term ΔV_TH_ (the V_TH_ difference between the after-stressing state and the initial state) in this study. It is worth noting that each ΔV_TH_ value was averaged over 3–6 devices. After 4500 s’ stressing, ΔV_TH_ of the unannealed device became 3.67 V. All the post-annealed devices exhibited better bias stress stability than the unannealed sample. It is interesting that the ΔV_TH_ value distinctively depended on the post-annealing atmosphere. As shown in Figure 3b, ΔV_TH_ decreased to 1.88 V, 2.50 V, 1.13 V and 1.38 V for the devices annealed in air, O_2_, N_2_ and vacuum, respectively. It is worth noting that the post-annealing treatments in N_2_ and vacuum showed much larger improvements than those in O_2_ and air.

**Figure 4:**
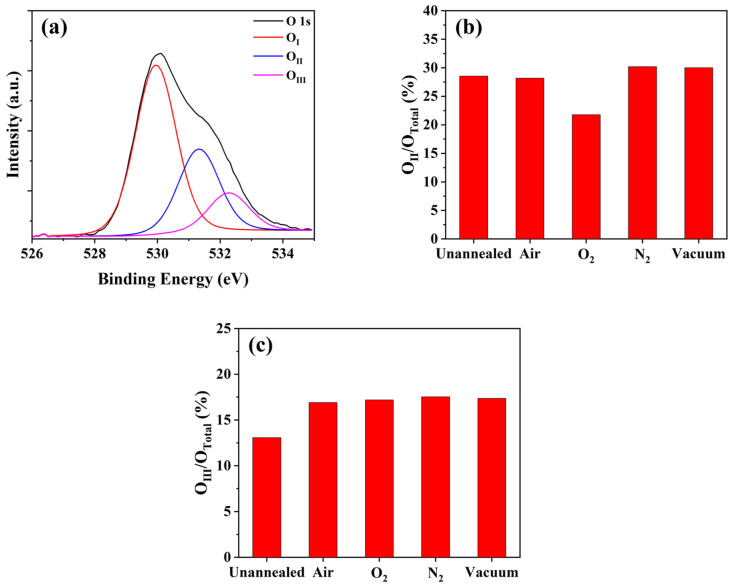
(**a**) Deconvolution diagram of the O1s peak of the unannealed a-IGZO films; the area ratios of (**b**) O_Ⅱ_ and (**c**) O_Ⅲ_ in the O1s peak of the a-IGZO films annealed under different atmospheres.

In order to determine the related physical mechanisms, we used the XPS technique to analyze the a-IGZO films (without or with passivation layers) annealed under various atmospheres. Firstly, we deposited 60 nm-thick a-IGZO films on glass substrates, annealed them under different atmospheres, and applied them to XPS measurements. Although these unpassivated films were different from the real situation in the TFT devices (see Figure 1), they could more evidently exhibit the influences of the annealing atmospheres on the back surfaces of the a-IGZO films. Figure 4a shows the deconvolution diagram of the O1s spectrum of the unannealed sample. The O1s peak can be deconvoluted into three sub-peaks using Gaussian fitting method which are approximately centered at 529.5 eV (O_I_), 530.9 eV (O_II_) and 532.0 eV (O_III_), respectively. The O_I_ peak represents the oxygen bonds with metal, the O_II_ peak is bound up with V_O_, and the O_III_ peak is related to the hydrated oxides defects [20,21]. Figure 4b,c show the peak area ratios of the O_II_ and O_III_ over the total area of O1s peak (O_Total_ = O_I_ + O_II_ + O_III_). The area ratio O_II_/O_Total_ of the a-IGZO films annealed under various atmospheres (unannealed, air, O_2_, N_2_ and vacuum) were 28.56%, 28.19%, 21.78%, 30.20% and 30.02%, respectively. One may observe that the post-annealing in O_2_ largely decreased the area ratio O_II_/O_Total_, whereas the heat treatments in air/N_2_/vacuum showed little effect in this regard. On the other side, the area ratio O_III_/O_Total_ of the a-IGZO films under different atmospheres (unannealed, air, O_2_, N_2_ and vacuum) were 13.08%, 16.91%, 17.19%, 17.53% and 17.37%, respectively. It is obvious that all the post-annealing treatments increased the area ratio O_III_/O_Total_.

**Figure 5:**
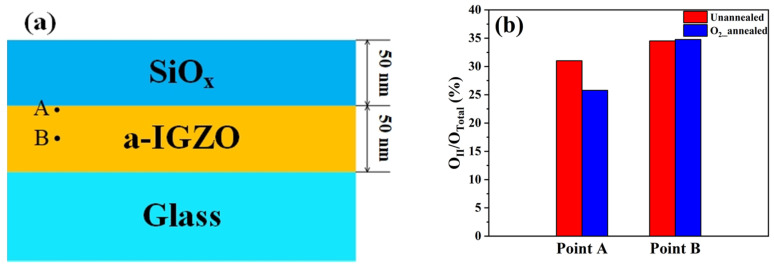
(**a**) The sample structure for the XPS depth profiling tests; (**b**) the area ratios of O_Ⅱ_ in the O1s peak of the unannealed and O_2__annealed samples.

As shown in Figure 4b, only O_2__annealing among five treatments evidently changed V_O_ in a-IGZO films, which are in need of much further study. Most importantly, could passivation layers prevent this effect? Attempting to address this question, we used XPS depth profiling technique to analyze the chemical bonding states in the a-IGZO films passivated by 50 nm-thick SiO_x_ [22]. As shown in Figure 5a, point A was exactly under the interface between the a-IGZO and SiO_x_, whereas point B was in the middle of a-IGZO films. The characterization results of the unannealed samples and the O_2__annealed samples are shown in Figure 5b. At point A, the area ratio O_II_/O_Total_ decreased from 31.0% to 25.8% by the O_2__annealing treatment, which was quite similar to the case of the unpassivated a-IGZO films (see Figure 4b). At point B, however, there was nearly no difference in the area ratio O_II_/O_Total_ between the unannealed (34.5%) and O_2__annealed (34.8%) samples. These results indicated that the oxygen atoms might also diffuse into the a-IGZO back surfaces and hence combine with V_O_ even if the SiO_x_ passivation layer was applied during the O_2__annealing treatments. Although the thickness of SiO_x_ used here (50 nm) was thinner than that of the passivation layer (200 nm) in the a-IGZO TFTs, we assume that this conclusion was also applied to the real devices.

**Figure 6:**
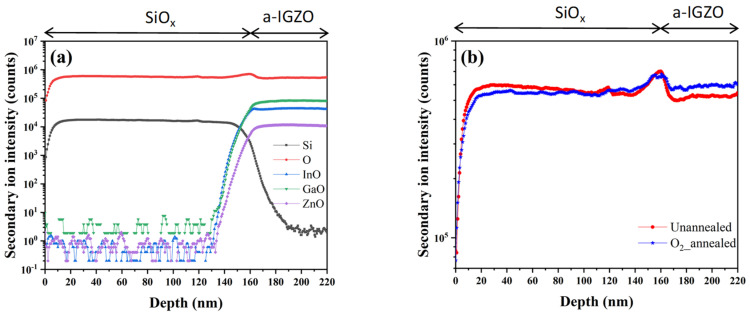
(**a**) SIMS depth profiles for the unannealed sample; (**b**) the oxygen depth distributions for the unannealed sample and the O_2__annealed sample.

In order to further ascertain the role of the ambient oxygen atoms during the thermal annealing treatments, we comparatively measured the SIMS profiles of the unannealed sample and O_2__annealed sample, both of which consisted of 60 nm-thick a-IGZO and 160 nm-thick SiO_x_ films. Here, the SIMS depth profiling technique was used to analyze the distribution of different elements in the unannealed and O_2_-annealed samples [23]. Figure 6a shows the depth dependence of the second ion intensity for the unannealed sample. One can observe that there were abrupt changes in Si, InO, GaO and ZnO at the depth of approximately 160 nm, which was close to the interfacial surface between SiO_x_ and a-IGZO. The O_2__annealing brought nearly the same SIMS picture as that of the unannealed sample except the data about oxygen ions. As shown in Figure 6b, the secondary ion intensity of oxygen remained almost unchanged within the SiO_x_ films by the O_2__annealing, implying that the entering oxygen atoms from the ambience during the annealing treatment in O_2_ were too few to be detected by SIMS. Therefore, we reasonably assume that the oxygen atoms entering the a-IGZO also could not be detected by SIMS. However, as shown in Figure 6b, the secondary ion intensity of the oxygen apparently increased for the O_2__annealed sample. We assume that the microstructure of the a-IGZO evidently changed during the annealing treatment in O_2_, which might be due to the entering oxygen atoms from the ambience although their concentration was quite small. This change also implied that the O_2__annealing might bring some oxygen-related-defects such as interstitial oxygen in the a-IGZO films [24,25].

**Figure 7:**
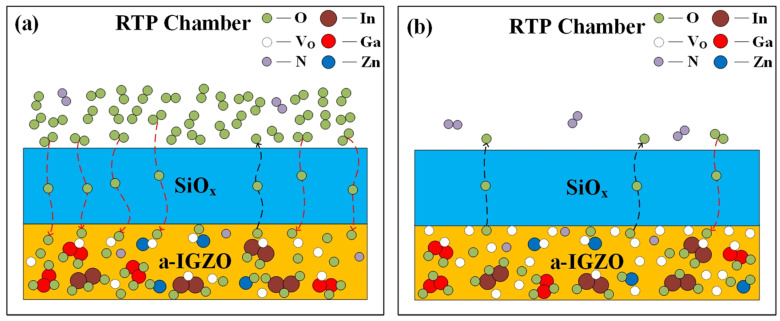
Schematic diagrams of the dynamic processes during the post-annealing treatments at 300 °C in (**a**) O_2_ (or air) and (**b**) vacuum (or N_2_).

It is well known that a-IGZO films are very sensitive to ambient atmospheres, especially oxygen and water [26,27]. For mass production, passivation layers are always used to isolate a-IGZO TFTs from the ambience. However, post-annealing treatments at high temperatures accelerate the interaction between the a-IGZO and outside world. This interaction is a dynamic process during annealing treatments. Here, we limit our discussion to the interactions between oxygen atoms because they are dominant in the electrical performance and stable properties of a-IGZO TFTs. Figure 7a shows the schematic diagram of the dynamic process during the annealing treatments at 300 °C in O_2_ (or air) atmospheres. In this situation, the oxygen atoms were full of the RTP chamber, so the oxygen diffusion from the chamber into the a-IGZO films should prevail that from the device channel layers into the ambience. It was reported that oxygen atoms could pass through dielectric films due to thermal diffusion at high temperatures [28,29]. Importantly, these newcomers (oxygen atoms) combined with oxygen vacancies and effectively decreased the V_O_ concentration in a-IGZO films, which was confirmed by the XPS measurement results (see Figure 4b and Figure 5b). This drop in the V_O_ concentration accordingly resulted in the larger V_TH_ values for the corresponding TFT devices, as shown in Figure 2b. It is worth noting that this effect was evidently weakened for the annealing treatments in air due to the existence of much more nitrogen atoms in the RTP chamber. Furthermore, the entering oxygen atoms also brought some oxygen-related-defects (see the discussion about the SIMS measurement results), and led to smaller μ_FE_ and larger I_OFF_ (as shown in Figure 2). Generally, heat treatments could improve the bias stress stability of a-IGZO TFTs due to the drops in the bulk defects and interface traps [30,31,32]; however, these oxygen-related-defects degraded this improvement effect. Specifically, the oxygen-related defects might make the V_O_ formation easier during positive bias stressing [33], which resulted in more unstable properties of a-IGZO TFTs. Thus, the PBS stability improvements for the a-IGZO TFTs annealed in O_2_ and air were not so evident, as shown in Figure 3b.

Figure 7b shows the dynamic process during the post-annealing at 300 °C in vacuum (or N_2_). In this situation, there were few oxygen atoms in the RTP chamber, so the oxygen diffusion from the a-IGZO film to the ambience was assumed to be dominant. If so, the V_O_ concentration would have become larger. However, this rise in the V_O_ concentration was not apparent due to the XPS measurement data. As shown in Figure 4b, the annealing in N_2_ (or vacuum) hardly changed the area ratio O_II_/O_Total_. We ascribe this phenomenon to the barrier effect of the SiO_x_ passivation layers. In contrast to Figure 7a, the SiO_x_ passivation layers effectively prevented the oxygen diffusion from the a-IGZO into the ambience during heat treatments in vacuum (or N_2_). Since the V_O_ concentration changed little in this case, the electron concentration in the channel layers of the a-IGZO TFTs remained nearly unchanged, leading to V_TH_ and μ_FE_ values similar to those of the unannealed device (see Figure 2b). As for the bias stress stability of the a-IGZO TFTs annealed in vacuum (or N_2_), we reasonably assume that there were no oxygen-related-defects included here, so the improvement effect over the stable properties of a-IGZO TFTs by heat treatments could be well kept [34]. As shown in Figure 3b, the annealing in vacuum (or N_2_) effectively improved the PBS stability of the a-IGZO TFTs with SiO_x_ passivation layers, which should be preferred in mass productions.

Finally, we briefly discussed two interesting questions relating this study. The first one is about the function of V_O_ and the other one is relating the choice of passivation materials. Some researchers reported the V_O_ drop led to better device performance [35,36], which is different from the results here. We ascribe this difference to the special effects of O_2__annealing treatments, i.e., they not only decreased the V_O_ concentration but also brought some oxygen-related-defects [24,25]. In this study, the SiO_x_ was selected as passivation layers, which was found to play important roles in the post-annealing treatments under different atmospheres. We assumed that other passivation materials should bring similar tendencies but to different extents. This assumption might be supported by the other research results from our group, which indicated that the a-IGZO TFTs with SiO_x_ and AlO_x_ passivation layers exhibited almost the same PBS stability if the different passivation layer thicknesses were used [33]. In fact, not only the passivation material but also some treatments on passivation layers might also influence their performance during heat treatments. It is reported that the thermal stability of TFTs can be improved by using fluorinated organic passivation, where the diffusion of F during the annealing process reduced the V_O_, leading to better thermal stability [35]. Although the different passivation layers showed somewhat different performances, all the above reports suggested that passivation layers have similar barrier effects during post-annealing treatments.

## 4. Conclusions

The post-annealing atmosphere effectively influenced the electrical performance and bias stress stability of the a-IGZO TFTs with SiO_x_ passivation layers. For the heat treatments in O_2_ (or air), the oxygen atoms in the ambience diffused into the a-IGZO and combined with oxygen vacancies, leading to less V_O_ and more oxygen-related defects; this resulted in larger V_TH_/I_OFF_, smaller μ_FE_ and the slightly better PBS stability of the a-IGZO TFTs. For the post-annealing process in vacuum (or N_2_), the oxygen diffusion was effectively prevented by the SiO_x_ passivation layers, and hence the V_O_ concentration hardly changed; the electrical performance of the a-IGZO TFTs showed nearly no change, but very importantly, their PBS stability evidently improved. This qualitative model was confirmed by the XPS and SIMS measurement results.

## Data Availability

The data presented in this study are available on request from the corresponding author.
